# Analysis of acupoint selection rules of acupuncture and moxibustion in ancient medical books for the treatment of somnolence: A review

**DOI:** 10.1097/MD.0000000000041676

**Published:** 2025-03-14

**Authors:** Hai-qing Zeng, Ze-dong-fang Yuan, Run-tian Wu, A-ling Tang, Zhi-sheng Huang

**Affiliations:** a Guangzhou University of Chinese Medicine, Guangzhou, China; b Guangzhou Hospital of Integrated Traditional and Western Medicine, Guangzhou, China; c Longhua Hospital Shanghai University of Traditional Chinese Medicine, Shanghai, China.

**Keywords:** acupuncture and moxibustion, data mining, rules of acupoint selection, somnolence, TCMICS

## Abstract

This study aimed to explore the rule of acupoint selection in the treatment of somnolence by ancient acupuncture and moxibustion. Using “Duomei” and other words as search terms in the Encyclopaedia of Traditional Chinese Medicine, a table containing elements such as literature sources, acupoint names, and frequency of acupoint selection was created. The table data was imported into the Traditional Chinese Medicine Inheritance Calculation Platform Software V3.5 (TCMICS) for statistical analysis to summarize the rules of acupoint selection. In the ancient medical books, the high-frequency acupoints of acupuncture and moxibustion in the treatment of somnolence were KI(Kidney)4(Dazhong)(24times,9.76%),KI3(Taixi)(23times,9.35%),LI(Large Intestine)3Sanjian(23times,9.35%) from high to low, and the high-frequency acupoints used for specific acupoints were Shu, Luo, and Bahui points from high to low. Association rule analysis showed that the core combination of high-frequency related acupoints was 3 groups, namely,“KI3(Taixi)KI6(Zhaohai)”(5times,2.03%),“KI4(Dazhong)HT(Heart)5(Tongli)”(5times,2.03%), and “LI3(Sanjian) ST(Stomach)45(Lidui) SJ(SanJiao)10(Tianjing)”(5times,2.03%). Cluster analysis showed that the effective cluster combination of acupoints was “LI3(Sanjian), LI2(Erjian),KI3(Taixi),ST45(Lidui),SJ10(Tianjing),KI6(Zhaohai),”“HT5(Tongli),KI4(Dazhong),”“RN(Ren) 12(Zhongwan),BL(Bladder)43(Gaohuang),ST36(Zusanli),RN6(Qihai).” In the ancient medical books, acupuncture and moxibustion was used to treat somnolence with distal point selection, and acupuncture and moxibustion prescriptions often used “KI3(Taixi)KI6(Zhaohai)” and other core acupoint combinations from the perspective of tonifying deficiency, relieving excess and giving consideration to both deficiency and excess, combined with acupoint selection and coordination based on syndrome differentiation and meridian differentiation, and focused on overall adjustment.

## 1. Introduction

“Duo mei,” which has the same meaning as “Duo wo” and “Duo mian” (These 3 words all have meanings similar to somnolence.), refers to a kind of disease characterized by frequent and constant sleepiness and increased actual sleep time.^[1–3]^ The name of “Duo mei” was first seen in the *Za Bing Yuan Liu Xi Zhu* written by ShenJin’ao in the Qing Dynasty: “Duo mei is a disease of the heart and spleen.” This has been related to somnolence in the *Yellow Emperor’s Canon of Internal Medicine*. This disease has affected people’s health since ancient times. Modern research shows that daytime sleepiness, which belongs to the category of somnolence, can be caused by sleep apnea hypopnea syndrome, insufficient sleep time, sleep rhythm disorder caused by night activities, adverse drug reactions, diabetes, trauma, infection, smoking and alcohol,^[4,5]^ and can increase the risk of accidents and diseases such as traumatic accidents, depression, hypertension.^[6–10]^ On the other hand, there is a certain scale of somnolence in people of different ages and occupations.^[3,11–15]^ Acupuncture therapy has been recorded in the *Yellow Emperor’s Canon of Internal Medicine* for the treatment of somnolence,^[16]^ and is still widely used by modern Traditional Chinese Medicine physicians for the treatment of a variety of diseases, including somnolence. However, the number of modern studies on acupuncture and moxibustion for the treatment of somnolence is small. What is more regrettable is that the idea of acupuncture and moxibustion point selection for this disease in ancient medical books has not been fully clarified. Therefore, the purpose of this study is to use data mining technology, based on the fifth edition series of *Encyclopedia of Traditional Chinese Medicine*, to sort out and analyze the relevant contents of ancient medical books on acupuncture and moxibustion treatment of somnolence, explore the rules of acupuncture and moxibustion point selection. Specifically, this includes the collection of acupoints and acupoint prescriptions recorded in ancient medical books for acupuncture and moxibustion treatment of somnolence, and the summary of more commonly used acupoints, the distribution characteristics of acupoints, the meridians to which the acupoints belong, the types of acupoints and the collocation of acupoints. In the end, this study will provide reference for modern clinical acupuncture and moxibustion treatment of somnolence.

## 2. Data and methods

### 2.1. Literature source

The fifth edition of the large-scale electronic series *Encyclopaedia of Traditional Chinese Medicine*, contains 1156 ancient medical books from all ages in China.

### 2.2. Determining search terms

**Figure 1.**
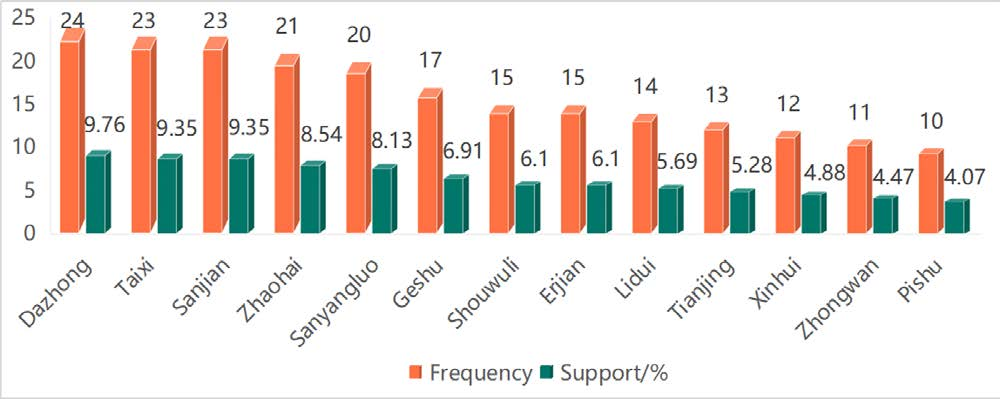
Bar chart for acupoints used for ≥10 times in ancient acupuncture and moxibustion prescriptions for somnolence. Annotation: Dazhong: KI4, Taixi: KI3, Sanjian: LI3, Zhaohai: KI6, Sanyangluo: SJ8, Geshu: BL17, Shouwuli: LI13, Erjian: LI2, Lidui: ST45, Tianjing: SJ10, Xinhui: DU22, Zhongwan: RN12, Pishu: BL20. The support was calculated by dividing the frequency of acupoints used by 246 total acupuncture and moxibustion prescriptions and multiplying the result by 100%.

**Figure 2.**
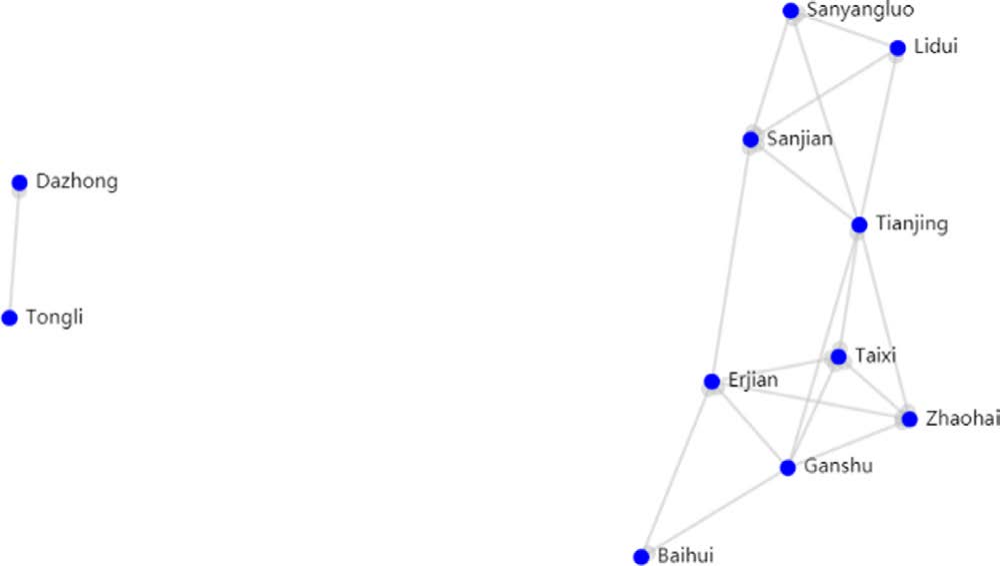
Acupoint Relationship Diagram with 3 Supports. Annotation: Dazhong: KI4, Tongli: HT5, Sanyangluo: SJ8, Lidui: ST45, Sanjian: LI3, Tianjing: SJ10, Taixi: KI3, Erjian: LI2, Zhaohai: KI6, Ganshu: BL18, Baihui: DU20.

**Figure 3.**
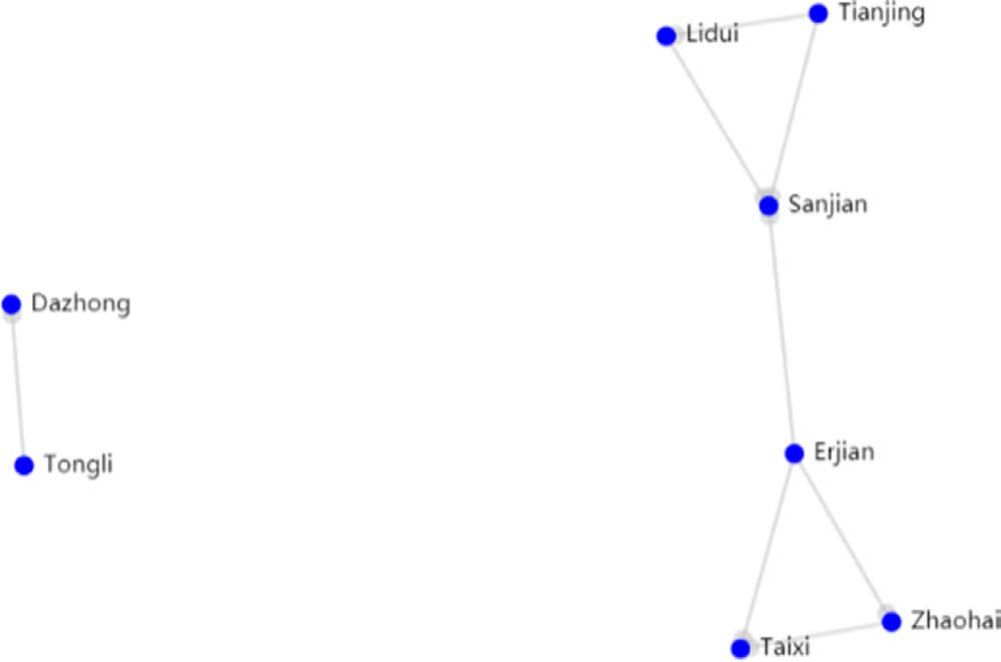
Acupoint Relationship Diagram with 4 Supports. Annotation: Dazhong: KI4, Tongli: HT5, Lidui: ST45, Tianjing: SJ10, Sanjian: LI3, Erjian: LI2, Taixi: KI3, Zhaohai: KI6.

**Figure 4.**
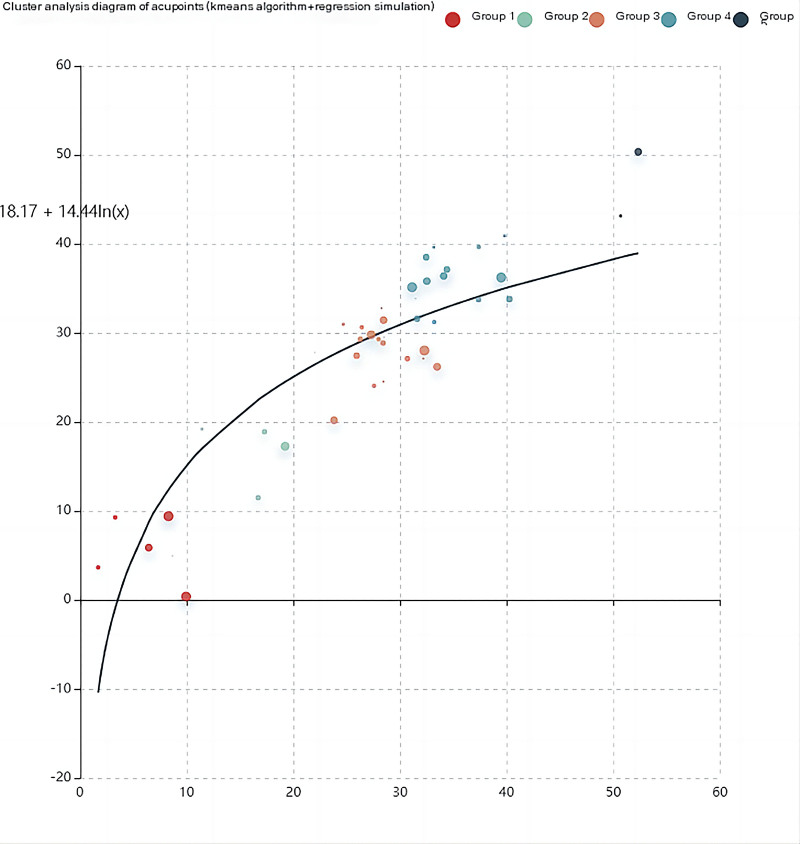
Cluster analysis of acupoint points (Cluster regression).

According to *the Dictionary of Traditional Chinese Medicine*, *Chinese Traditional Medicine and Materia Medica Subject Headings* and *the Terms of Clinical Diagnosis and Treatment of Traditional Chinese Medicine*, the names and symptoms of somnolence were standardized, and 8 terms were identified as literature search terms, including “Duo mei,” “Shi wo,” “Shi shui,” “Duo mian,” “Shan mian,” “Duo wo,” “Duo shui” and “Dan yu mei” (These search terms all have meanings similar to somnolence; see Figures 1-9, Supplemental Digital Content, http://links.lww.com/MD/O468,which demonstrate search term sources from authoritative literature). Set the search scope to “text search,” and only select one of the 8 search terms for each search. Based on the above word search, the context content was further consulted to check for omissions and compensate for deficiencies. To ensure the integrity of the search results, if the search words have interchangeable words, but the word meaning is the same as the above search words, they are retrieved as search words.

### 2.3. Inclusion criteria

Prescriptions with acupuncture and moxibustion as the treatment methods, including the single acupuncture treatment prescription, the single moxibustion treatment prescription, and the combined prescription of acupuncture and moxibustion treatment, and including a single acupoint prescription and multiple acupoint prescriptions. Prescriptions with specific names of selected acupoints recorded, detailed descriptions of the location of selected acupoints without specific names of selected acupoints recorded, and prescriptions corresponding to known acupoints can be included by virtue of location description. In case of duplication due to the quotation of previous medical records by later medical records, only the original medical records will be included; if there are medical books that have their own different viewpoints on acupoint selection while quoting the contents of previous medical books, they will also be included. When the same acupoint selection prescription is retrieved by different search terms, all the same prescriptions are included. The bibliography of acupoint selection sources was written before the end of the Qing Dynasty.

### 2.4. Exclusion criteria

Prescriptions not involving acupoint selection. When the same prescription appears in different positions in the same medical record, it is treated as one result without repeated entries. If the relevant information of the selected acupoints is insufficient to determine the prescription of specific selected acupoints, it will not be entered. Prescription of acupuncture combined with non-acupuncture therapies.

### 2.5. Data screening and entry

Two researchers jointly completed the data screening and input. If there was disagreement on the disease name and specific acupoints, consult and discuss with the third researcher to clarify the results and reach a consensus. After collecting and summarizing the data, the medical book name, acupoint name, acupoint meridians, acupoint distribution location, specific acupoints, use of acupuncture and moxibustion and other elements were entered into a table, the original database was established, and the data were imported into the Traditional Chinese Medicine Inheritance Computing platform.

### 2.6. Statistical treatment

Using the v3.5 software of Traditional Chinese Medicine Inheritance Computing platform, the frequency analysis method was used to carry out the frequency statistics of the acupuncture point selection prescription data that met the requirements, and the association rule analysis, cluster analysis and other statistical analysis were carried out. Among them, association analysis uses the “acupoint mode” and “rule analysis”(Analysis of association rules for acupoints based on FP-Tree algorithm) functions in the “acupoint combination” function of the platform, which are realized by setting the appropriate number of supports(the minimum number of occurrences of a certain acupoint combination in the total acupoint prescription), confidence(the probability that one acupoint and another acupoint combination appear together in the same acupuncture prescription), and FP-Tree Optimization Algorithm. The analysis method involves scanning the data, obtaining the count of all frequent item sets, removing items whose support is lower than the threshold, and then placing a frequent set into the item header table in descending order of support. Subsequently, the sorted dataset was read in and inserted into the FP tree. The nodes at the top of the sorting were set as ancestor nodes, and the nodes at the bottom were set as descendants. When shared ancestor nodes exist, the count of shared ancestor nodes increases by one. If a new node appears after insertion, the corresponding node in the item header table is linked to the new node through a node-linked list. When all data are inserted into the FP tree, a tree is established. Finally, from the bottom of the item header table, we find the corresponding conditional pattern base of the item header table item in ascending order, and then recursively mine the frequent itemsets of the item header table items from the conditional pattern base. When the number of items in the frequent itemset is not limited, all previous frequent itemsets are returned. If the number of items is limited, the frequent itemsets that meet the requirements for a limited number of items, that is, complete the analysis. (More specific settings and calculations are completed by the software itself.); Cluster analysis uses the “core combination” and “cluster regression” functions in the “acupoint clustering” function of the platform, which are realized by K-means algorithm and linear regression and other regression models. The algorithm process involves randomly selecting k points in the sample as cluster centers, calculating the distance between other samples in the sample and k cluster centers, and using these samples as the category closest to the cluster center. Then, the average value for each category of the classified samples is calculated, the new cluster centroid is obtained, and it is compared with the k cluster centroids obtained from the previous calculation. If the cluster centroid changes, the distance between other samples and the k cluster centers is repeated and classified according to the cluster center steps. If the calculation is stopped, and the clustering results are obtained. Finally, the results are derived, summarized and discussed.

## 3. Results

### 3.1. Literature screening results

The preliminary search yielded 5217 results (The search term “Duo mei” yielded 91 results, “Shi wo” yielded 2308 results, “Shi shui” yielded 78 results, “Duo mian” yielded 468 results, “Shan mian” yielded 47 results, “Duo wo” yielded 411 results, “Duo shui” yielded 1204 results and “Dan yu mei” yielded 610 results.). According to the inclusion and exclusion criteria, 246 acupuncture acupoint selection prescriptions were summarized, involving 45 acupoints, including 218 single-acupoint prescriptions and 28 multi-acupoint compound prescriptions. The total frequency of acupoint use was 314 times.

### 3.2. Analysis of acupoint use frequency

There were 13 acupoints with a frequency of more than 10 times (see Article 10, Supplemental Digital Content, http://links.lww.com/MD/O469, which demonstrates reasons for Setting the minimum acupoint citation frequency to 10) in the treatment of somnolence by acupuncture and moxibustion, of which 6 were ranked in the top 5, followed by KI4(Dazhong) (24times), KI3(Taixi) (23times), LI3(Sanjian) (23times), KI6(Zhaohai) (21times), SJ8(Sanyangluo) (20times), and BL17(Geshu) (17times), the other high-frequency acupoints includeLI13(Shouwuli) (15times), LI2(Erjian) (15times), ST45(Lidui) (14times), SJ10(Tianjing) (13times), DU(Du) 22(Xinhui) (12times), RN12(Zhongwan) (11times), and BL20(Pishu) (10times), as shown in Figure 1.

### 3.3. Acupoint meridian analysis

Thirteen meridians were involved in the selection of acupoints for acupuncture and moxibustion treatment for somnolence. The 5 meridians with the highest frequency of use were Foot Shaoyin Kidney meridian (75 times, 23.89%), Hand Yangming Large Intestine meridian (59 times, 18.79%), Foot Taiyang Bladder meridian (38 times, 12.10%), Hand Shaoyang Sanjiao meridian (33 times, 10.51%), and Du meridian (21 times, 6.69%). The cumulative frequency of use of the 5 meridians was 228 (72.61%), and the cumulative number of points was 22 (48.89%) while the hand Taiyang small intestine meridian’s acupoints were only used once and were rarely used; Hand Jueyin pericardium meridian’s acupoints were not selected and 3 extra nerve points, including Taiyinqiao, Bijiao, and Tongguan, were used a total of 8 times (2.55%). Table 1 lists the results.

**Table 1:** Frequency analysis of acupoint meridian tropism of ancient acupuncture and moxibustion prescriptions for somnolence

Serial number	Meridians	Frequency of use		Number of acupoints		Acupoint selection and frequency of acupoint use
		Frequency	Percentage/%	Number	Percentage/%	
1	Kidney meridian of foot Shaoyin	75	23.89	6	13.33	KI4(Dazhong)(24),KI3(Taixi)(23),KI6(Zhaohai)(21),KI1(Yongquan)(5),KI2(Rangu) (1), KI7(Fuliu) (1)
2	Hand Yangming large intestine meridian	59	18.79	4	8.89	LI3(Sanjian)(23),LI13(Shouwuli) (15),LI2(Erjian)(15),LI12(Zhouliao) (6)
3	Foot Taiyang bladder meridian	38	12.10	8	17.78	BL17(Geshu) (17), BL20(Pishu) (10),BL18(Ganshu)(4),BL23(Shenshu) (2), BL43(Gaohuang) (2), BL58(Feiyang)(1), BL13(Feishu) (1), BL19(Danshu) (1)
4	Three jiao meridians of hand Shaoyang	33	10.51	2	4.44	SJ8(Sanyangluo)(20),SJ10(Tianjing) (13)
5	Du meridian	21	6.69	2	4.44	DU22(Xinhui)(12),DU20(Baihui) (9)
6	Stomach meridian of Foot Yangming	19	6.05	4	8.89	ST45(Lidui)(14),ST41(Jiexi) (3), ST36(Zusanli) (1), ST31(Biguan) (1)
7	Spleen meridian of foot Taiyin	15	4.78	3	6.67	SP5(Shangqiu)(9),SP4(Gongsun) (4), SP6(Sanyinjiao) (2)
8	Ren channel	12	3.82	2	4.44	RN12(Zhongwan)(11),RN6(Qihai) (1)
9	Liver meridian of foot Jueyin	10	3.18	2	4.44	LR10(Zuwuli)(9),LR13(Zhangmen) (1)
10	Lung meridian of hand Taiyin	9	2.87	4	8.89	LU3(Tianfu) (6),LU7(Lieque)(1), LU1(Zhongfu)(1),LU11(Shaoshang) (1)
11	Extra meridian acupoint	8	2.55	3	6.67	Tai Yin Qiao (4), Bijiao (3), and Tongguan (1)
12	Gallbladder meridian of foot Shaoyang	7	2.23	3	6.67	GB39(Xuanzhong)(3),GB38(Yangfu) (3), GB24(Riyue) (1)
13	Hand Shaoyin heart meridian	7	2.23	1	2.22	HT5(Tongli) (7)
14	Hand Taiyang small intestine meridian	1	0.32	1	2.22	SI13(Quyuan) (1)

BL = bladder, DU = du, GB = gallbladder, HT = heart, KI = kidney, LI = large intestine, LR = liver, LU = lung, PC = pericardium, RN = ren, SI = small intestine, SJ = SanJiao, SP = spleen, ST = stomach.

### 3.4. Analysis of acupoint distribution

The selected acupoints for acupuncture treatment of somnolence were mainly concentrated in the lower and upper limbs, and the rest of the head, shoulder, back, waist, chest, and abdomen were also distributed. Based on frequency, the selected acupoints were arranged from high to low as follows: lower limbs (130 times, 41.40%), upper limbs (107 times, 34.08%), shoulder and back (36 times, 11.46%), head (24 times, 7.64%), abdomen (13 times, 4.14%), chest (2 times, 0.64%), and waist (2 times, 0.64%), as shown in Table 2.

**Table 2:** Distribution of acupoints used in ancient acupuncture for treating somnolence

Serial number	Parts of the body	Frequency	Number of acupoints	Acupoints and their frequency of use
1	lower limbs	130	19	KI4(Dazhong)(24),KI3(Taixi)(23),KI6(Zhaohai)(21),ST45(Lidui)(14),SP5(Shangqiu)(9),LR10(Zuwuli)(9),KI1(Yongquan)(5),Taiyinqiao(4),SP4(Gongsun)(4),ST41(Jiexi)(3),GB39(Xuanzhong)(3),GB38(Yangfu)(3),SP6(Sanyinjiao)(2),KI2(Rangu)(1),ST36(Zusanli)(1),ST31(Biguan)(1),BL58(Feiyang)(1),KI7(Fuliu)(1),Tongguan(1)
2	upper limb	107	10	LI3(Sanjian) (23), SJ8(Sanyangluo) (20)LI13(Shouwuli) (15), LI2(Erjian)(15), SJ10(Tianjing) (13), HT5(Tongli) (7)LU3(Tianfu) (6)LI12(Zhouliao) (6)LU7(Lieque) (1)LU11(Shaoshang) (1)
3	shoulder and back	36	7	BL17(Geshu) (17), BL20(Pishu) (10), BL18(Ganshu) (4), BL43(Gaohuang) (2), SI13(Quyuan) (1), BL13(Feishu) (1), BL19(Danshu) (1)
4	head	24	3	DU22(Xinhui) (12), DU20(Baihui) (9), Bijiao (3)
5	abdomen	13	3	RN12(Zhongwan) (11), LR13(Zhangmen) (1), RN6(Qihai) (1)
6	chest	2	2	LU1(Zhongfu) (1), GB24(Riyue) (1)
7	waist	2	1	BL23(Shenshu) (2)

BL = bladder, DU = du, GB = gallbladder, HT = heart, KI = kidney, LI = large intestine, LR = liver, LU = lung, PC = pericardium, RN = ren, SI = small intestine, SJ = SanJiao, SP = spleen, ST = stomach.

### 3.5. Analysis of specific acupoints

Among the 45 acupoints used in acupuncture treatment for somnolence, 30 acupoints were specific acupoints, and the proportion of specific acupoints reached 66.67%. Except for the Xi acupoint, which was not selected, the other 12 types of acupoints were selected to varying degrees. Based on the frequency of use, the 12 types of specific acupoints used were ranked as follows: Shu acupoint (46 times, 17.49%), Luo acupoint (37 times, 14.07%), Bahui acupoint (32 times, 12.17%), Bamai intersection acupoint (26 times, 9.89%), Yuan acupoint (23 times, 8.75%), Jing acupoint (20 times, 7.60%), Beishu acupoint (18 times, 6.84%), Ying acupoint (16 times, 6.08%), meridians (16 times, 6.08%), He acupoint (14 times, 5.32%), Mu acupoint (14 times, 5.32%), and Xiahe acupoint (1 time, 0.38%), as shown in Table 3.

**Table 3:** Use of ancient acupuncture and moxibustion on specific acupoints of somnolence

Serial number	Specific acupoints	Frequency	Number of acupoints	Acupoints and their frequency of use
1	Shuxue	46	2	LI3(Sanjian) (23), KI3(Taixi) (23)
2	Luoxue	37	5	KI4(Dazhong) (24), HT5(Tongli) (7), SP4(Gongsun) (4), BL58(Feiyang) (1), LU7(Lieque) (1)
3	Bahuixue	32	4	BL17(Geshu)(1), RN12(Zhongwan) (11), GB39(Xuanzhong) (3), LR13(Zhangmen) (1)
4	Bamaijiaohuixue	26	3	KI6(Zhaohai) (21), SP4(Gongsun) (4), LU7(Lieque) (1)
5	Yuanxue	23	1	KI3(Taixi) (23)
6	Jingxue	20	3	ST45(Lidui) (14), KI1(Yongquan) (5), LU11(Shaoshang) (1)
7	Beishuxue	18	5	BL20(Pishu) (10), BL18(Ganshu) (4), BL23(Shenshu) (2), BL13(Feishu) (1), BL19(Danshu) (1)
8	Yingxue	16	2	LI2(Erjian) (15), KI2(Rangu) (1)
9	Jingxue	16	4	SP5(Shangqiu) (9), ST41(Jiexi) (3), GB38(Yangfu) (3), KI7(Fuliu) (1)
10	Hexue	14	2	SJ10(Tianjing) (13), ST36(Zusanli) (1)
11	Muxue	14	4	RN12(Zhongwan) (11), LU1(Zhongfu) (1), GB24(Riyue) (1), LR13(Zhangmen) (1)
12	Xiahexue	1	1	ST36(Zusanli) (1)
13	Xixue	0	0	

BL = bladder, DU = du, GB = gallbladder, HT = heart, KI = kidney, LI = large intestine, LR = liver, LU = lung, PC = pericardium, RN = ren, SI = small intestine, SJ = SanJiao, SP = spleen, ST = stomach.

### 3.6. Association rule analysis of acupoint compatibility

Through the Traditional Chinese Medicine Inheritance Computing Platform, compatibility association analysis (based on the FP-tree algorithm) was performed on 246 summarized acupuncture and moxibustion prescriptions and 45 points among them. In specific operations, we set the number of support points to 3, which means that the combination of acupoints should appear in at least 3 prescriptions at the same time with a confidence level of 0.8, which means that the credibility of the association rules is above 80%. There were a total of 31 acupoint combinations and 32 association rules, including 11 acupoints, as shown in Figure 2; If the number of support points was set to 4 and the confidence level was still set to 0.8, there were a total of 10 acupoint combinations and 6 association rules, including 8 acupoints, as shown in Figure 3. Based on the analysis of the 2 different settings, the acupoint combinations were arranged in order of frequency and association rules in descending order of confidence, as shown in Tables 4 and 5.

**Table 4:** Core acupoint combinations of ancient acupuncture and moxibustion for somnolence

Serial number	Acupoint combination	Frequency	Serial number	Acupoint combination	Frequency
1	Taixi-Zhaohai	5	17	Taixi-Zhaohai-Tianjing	3
2	Dazhong-Tongli	5	18	Sanyangluo-Lidui	3
3	Lidui-Tianjing	5	19	Sanjian-Sanyangluo-Tianjing	3
4	Sanjian-Lidui-Tianjing	5	20	Taixi-Zhaohai-Tianjing-Ganshu	3
5	Sanjian-Lidui	5	21	Taixi-Tianjing-Ganshu	3
6	Sanjian-Tianjing	5	22	Taixi-Tianjing	3
7	Taixi-Erjian	4	23	Erjian-Baihui	3
8	Sanjian-Erjian	4	24	Erjian-Ganshu	3
9	Zhaohai-Erijian	4	25	Erjian-Baihui-Ganshu	3
10	Taixi-Zhaohai-Erjian	4	26	Sanjian-Sanyangluo-Lidui	3
11	Sanjian-Sanyangluo	3	27	Zhaohai-Ganshu	3
12	Taixi-Ganshu	3	28	Sanjian-Sanyangluo-Lidui-Tianjing	3
13	Baihui-Ganshu	3	29	Sanyangluo-Tianjing	3
14	Zhaohai-Tianjing	3	30	Tianjing-Ganshu	3
15	Zhaohai-Tianjing-Ganshu	3	31	Taixi-Zhaohai-Ganshu	3
16	Sanyangluo-Lidui-Tianjing	3			

Taixi: KI3, Zhaohai: KI6, Dazhong: KI4, Tongli: HT5, Lidui: ST45, Tianjing: SJ10, Sanjian: LI3, Erjian: LI2, Sanyangluo: SJ8, Ganshu: BL18, Baihui: DU20.

**Table 5:** Association rules of acupoint combinations in ancient acupuncture treatment of somnolence

Serial number	Association rules	Confidence
1	Lidui,Tianjing→Sanjian	1
2	Sanjian, Tianjing→Lidui	1
3	Sanjian,Lidui→Tianjing	1
4	Tianjing,Ganshu→Zhaohai	1
5	Zhaohai,Ganshu→Tianjing	1
6	Zhaohai,Tianjing→Ganshu	1
7	Sanyangluo,Tianjing→Lidui	1
8	Sanyangluo,Lidui→Tianjing	1
9	Zhaohai,Tianjing→Taixi	1
10	Taixi,Tianjing→Zhaohai	1
11	Sanyangluo,Tianjing→Sanjian	1
12	Sanjian,Sanyangluo→Tianjing	1
13	Zhaohai,Tianjing,Ganshu→Taixi	1
14	Taixi,Tianjing,Ganshu→Zhaohai	1
15	Taixi,Zhaohai,Ganshu→Tianjing	1
16	Taixi,Zhaohai,Tianjing→Ganshu	1
17	Tianjing,Ganshu→Taixi	1
18	Taixi,Ganshu→Tianjing	1
19	Taixi,Tianjing→Ganshu	1
20	Baihui,Ganshu→Erjian	1
21	Erjian,Ganshu→Baihui	1
22	Erjian,Baihui→Ganshu	1
23	Sanyangluo,Lidui→Sanjian	1
24	Sanjian,Sanyangluo→Lidui	1
25	Sanyangluo,Lidui,Tianjing→Sanjian	1
26	Sanjian,Sanyangluo,Tianjing→Lidui	1
27	Sanjian,Sanyangluo,Lidui→Tianjing	1
28	Zhaohai,Erjian→Taixi	1
29	Taixi,Erjian→Zhaohai	1
30	Taixi,Zhaohai→Erjian	0.8
31	Zhaohai,Ganshu→Taixi	1
32	Taixi,Ganshu→Zhaohai	1

Lidui: ST45, Tianjing: SJ10, Sanjian: LI3, Ganshu: BL18, Zhaohai: KI6, Sanyangluo: SJ8, Taixi: KI3, Erjian: LI2, Baihui: DU20.

### 3.7. Cluster analysis of acupoints

Using the “acupoint clustering” function in the Traditional Chinese Medicine Inheritance and Calculation Platform, we conducted cluster analysis on the 28 acupuncture and moxibustion acupoint prescriptions for somnolence, combined with the main diseases and efficacy of acupoints, and adopted kmeans algorithm + clustering + regression. Combining the effectiveness of the k value and TCM significance of grouping acupoint combinations, the final setting of the k value was 5, and 5 core acupoint combinations were obtained, as shown in Table 6. Figure 4 shows a simulation of acupoint clustering regression. Among them, the different color points in the regression graph, that is, the closer the different categories of acupuncture and moxibustion acupoint selection prescriptions are to the regression curve, the closer the category of prescriptions is to the category of core acupoints. Thus, it can be seen that the category 3 and category 4 acupoint prescriptions are closer to the core acupoints, and the other categories are slightly farther.

**Table 6:** Core combination of cluster analysis

Classification number	Core acupoint combination	Number
1	Sanjian,Erjian,Taixi,Lidui,Tianjing,Zhaohai	14
2	Geshu,Tongli,Biguan,Dazhong,Zhongfu,Shouwuli	4
3	Ganshu,Sanyinjiao,Pishu,Yongquan,Jiexi,Zhaohai	4
4	Tongli,Dazhong	4
5	Zhongwan,Gaohuang,Zusanli,Qihai	2

Sanjian: LI3, Erjian: LI2, Taixi: KI3, Lidui: ST45, Tianjing: SJ10, Zhaohai: KI6, Geshu: BL17, Tongli: HT5(Tongli), Biguan: ST31, Dazhong: KI4, Zhongfu: LU1, Shouwuli: LI13, Ganshu: BL18, Sanyinjiao: SP6, Pishu: BL20, Yongquan: KI1, Jiexi: ST41, Zhongwan: RN12, Gaohuang: BL43, Zusanli: ST36, Qihai: RN6.

## 4. Discussion

### 4.1. Analysis of acupoint frequency

According to the results of the above analysis of the frequency of use of acupoints, 13 acupoints with a frequency ≥ 10 times can be regarded as the selection of acupuncture and moxibustion points for the treatment of somnolence in ancient medical books. The most frequently used acupoint KI4(Dazhong) is used to treat diseases including somnolence.^[17]^Some studies have also shown that the use of Needle Warming Moxibustion at KI4(Dazhong) has a certain effect on improving the cognitive function of dementia patients with insufficiency of marrow-sea(A type of disease believed by Traditional Chinese Medicine, the mechanism of which is the insufficient marrow in the human body, leading to symptoms such as decreased intelligence, fatigue, and soreness in the waist and knees.), which is similar to the wake-up effect of Traditional Chinese Medicine.^[18]^ At the same time, wake-up is also one of the effects needed for the treatment of somnolence; The LI2(Erjian), LI3(Sanjian), and LI13(Shouwuli) acupoints belong to the Large Intestine Meridian of Hand-yangming, and are now commonly used to treat heat and pain syndromes(Heat syndrome refers to types of diseases characterized by hyperactivity of human body functions; Pain syndrome refers to types of diseases characterized by various types of pain as the main manifestation.). However, in ancient literature, somnolence is also one of their main treatment symptoms, just as the *A-B Classic of Acupuncture and Moxibustion* records: “Sleeping a lot, feeling shoulder pain and cold... LI2(Erjian) can be used to treat these diseases.” “Sleeping more, Feeling full in the chest and frequently hearing bowel sounds, LI3(Sanjian) can be used to treat these diseases.” “People who sleep too much and do not want to move their limbs or have a yellowish body can be treated with moxibustion at the LI13(Shouwuuli)”;^[19]^ Both the KI3(Taixi) and KI6(Zhaohai) acupoints, which belong to the same kidney meridian as the KI4(Dazhong) acupoint, are used to treat somnolence. Combined with the opposite or adverse effects of somnolence caused by insomnia, as well as the bidirectional regulatory effect of acupoints,^[20]^ it can be concluded that both acupoints can also treat somnolence. In *Pujifang Acupuncture and Moxibustion*, the 3 acupoints of the Kidney Channel mentioned above are all placed in the same prescription for the treatment of somnolence,^[21]^ which is also a powerful evidence for the treatment of somnolence with the 2 acupoints; The 2 acupoints of Sanjiao meridian, SJ8(Sanyangluo) and SJ10(Tianjing) are also used as acupoints for treating somnolence in the *A-B Classic of Acupuncture and Moxibustion,* and they are also used to treat diseases such as pain and deafness.^[19]^ Deafness, in Traditional Chinese Medicine theory, is often caused by kidney qi deficiency (similar to renal dysfunction). The kidney opens the orifices in the ears and 2 yin (Traditional Chinese Medicine believes that the relationship between the kidneys, ears, reproductive organs, and anus is relatively close), and when the essence of the kidney is insufficient (similar to renal dysfunction), it can easily lead to deafness.^[22]^ And It also often causes the manifestation of “wanting to sleep” in Shaoyin’s disease (A type of disease believed by Traditional Chinese Medicine to be mainly caused by weak heart and kidney deficiency, characterized by fatigue, fear of cold, and weak pulse), which belongs to the category of somnolence. Therefore, the 2 acupoints of the Sanjiao meridian are used as auxiliary acupoints to treat the deafness symptoms of somnolence; A modern research has shown that acupuncture at the acupoints of BL17(Geshu) and 5 Organs (Heart, liver, spleen, lung, and kidney) has good therapeutic effects on insomnia,^[23]^ and the high-frequency acupoint of BL20(Pishu) is one of the 5 organs acupoints. Combined with the bidirectional regulatory effect of these acupoints, it can be considered that the combination of these 2 acupoints can have a certain therapeutic effect on somnolence; The acupoint of Ren Meridian, RN12(Zhongwan), is a commonly used acupoint for regulating the function of spleen and stomach, and is also used to treat insomnia. Combined with its bidirectional regulatory function, it can exert therapeutic effects on somnolence; ST45(Lidui) is often used as an acupoint for treating heat and pain syndromes. When somnolence is caused by heat pathogenic factors damaging yin(A type of Traditional Chinese Medicine syndrome, which refers to the burning nature of a disease that consumes substances such as water and fluids in the human body), gallbladder heat or hepatic excess(A type of Traditional Chinese Medicine syndrome that refers to the state of hyperactivity and heat in both the liver and gallbladder),^[24]^ it can be used for clearing heat and purging fire(Inhibit the diseases of the 2 hot states mentioned above). There are also studies showing that using the penetrating needling method to needle the DU22(Xinhui) and DU21(Qianding), which have similar effects, can significantly promote the improvement of cerebral hemodynamics and the recovery of neurological function in patients with cerebral infarction.^[25]^ This therapeutic effect is consistent with the awakening effect required for treating somnolence mentioned earlier.

### 4.2. Analysis of meridian usage

From the frequency of use of meridians, the Foot Shaoyin Kidney Meridian, Hand Yangming Large Intestine Meridian, Foot Taiyang Bladder Meridian, Hand Shaoyang San Jiao Meridian, and Governor Meridian are commonly used in ancient medical texts to treat somnolence. The Foot Shaoyin Kidney Meridian, as mentioned in the *Yellow Emperor’s Canon of Internal Medicine*, has a therapeutic function for “excessive sleep”: “It is responsible for treating patients with kidney diseases, such as hot mouth, dry tongue... collapse, excessive sleep, and pain under the feet.”^[16]^ It can be seen that it has a close relationship with somnolence for a long time and has a therapeutic effect on somnolence. The Hand Yangming Large Intestine Meridian has been shown to have symptoms such as stroke and hemiplegia as diseases that it can mainly be used to treat. It functions to awaken the brain and regulate the mind,^[26]^ which is also in line with one of the treatment principles of somnolence; The Foot Taiyang Bladder Meridian has been shown to have a clear therapeutic effect on chronic fatigue syndrome, which is mainly characterized by somnolence, through the corresponding acupoints of the 5 organs on the back.^[27]^ In addition, this meridian has the same therapeutic effect as the Three Foot Yang Meridians(Bladder meridian, gallbladder meridian, and stomach meridian) on mental disorders.^[17]^ Therefore, its therapeutic effect on somnolence is clear; The San Jiao Meridian of Hand Shaoyang has been shown to have a good therapeutic effect on insomnia by needling its acupoints.^[28]^ Based on the bidirectional regulation mechanism of acupoints, combined with the close connection between insomnia and somnolence, both diseases belong to the category of mental disorders, it can be inferred that it also has a certain therapeutic effect on somnolence; The governor meridian, as recorded in the *Yellow Emperor’s Canon of Internal Medicine*, “Distributed upwards in a straight line from the lower abdomen, passing through the center of the navel and the upper heart.,”^[16]^ while *Classic on medical problems* also records that “It goes up to the DU16(Fengfu) and belongs to the Brain.”^[29]^ its meridians pass through the heart, brain, and other organs that are closely related to somnolence, and both the governor meridian and Ren meridian’s acupoints have the effect of treating mental disorders. Combined with some studies, it has been shown that it has a strong relationship with paroxysmal sleeping sickness, which belongs to the somnolence category. It is worth paying attention to the therapeutic effect of the governor meridian on somnolence.^[30]^ It is also easy to understand that this acupoint is commonly used for acupuncture and moxibustion treatment of somnolence.

### 4.3. Analysis of acupoint distribution

From the acupoint selection and distribution of acupuncture and moxibustion for the treatment of somnolence, the acupoint selection for the treatment of somnolence is mainly distributed in the upper and lower limbs, while the acupoint distribution and frequency of use in other parts of the body are significantly lower. Somnolence is closely related to the mind, with the heart governing the mind and brain being the home of the primordial spirit (Traditional Chinese Medicine believes that human consciousness is controlled by the heart, and the brain is the place where consciousness stays.). Therefore, the disease location mostly belongs to the heart and brain. It will be seen from this that the idea of selecting acupoints mainly based on the limbs is more in line with the principle of selecting acupoints from the distal end, as stated in the *Yellow Emperor’s Canon of Internal Medicine*: “If the disease is on the upper part, it should be taken from the lower part; If the disease is in the lower part, it should be taken from the upper part; Diseases in the head should be treated from the feet.”^[16]^ According to the “Genjiebiaoben” theory (The theory of meridians in Traditional Chinese Medicine refers to the close connection between the movement of qi in the meridians and the upper and lower parts, as well as the internal and external parts of the human body.), the limbs are the root of the meridians, that is, the place where the qi of the meridians starts and concentrates, and have a therapeutic effect on the place where the qi of the meridians located in the head and body comes down and spreads,^[17]^ this is also one of the supporting evidence for remote acupoint selection; Outside of the limbs, more acupoints are selected for the shoulder, back, head, and abdomen, while fewer are selected for chest, waist. This selection of acupoints is also closer to the location of somnolence disease, that is, the local acupoint selection approach. Therefore, somnolence’s acupoint selection approach is mainly based on distal acupoints, combined with certain local acupoints.

### 4.4. Specific acupoint frequency analysis

In ancient times, the top 3 selected specific acupoints for the treatment of somnolence by acupuncture and moxibustion were Shu acupoints, Luo acupoints and Bahui acupoints according to the frequency from high to low. Studies have shown that acupuncture at Shu acupoints such as LR3(Taichong), KI3(Taixi), and SP3(Taibai) has a positive effect on improving cognitive function,^[31]^ which corresponds to the effect of TCM in awakening the mind and opening the orifices(The therapy in Traditional Chinese Medicine that awakens patients from a coma), which is one of the treatment principles for somnolence.^[32]^ Modern research has also shown that acupuncture at Luo acupoints such as HT5(Tongli) can activate language related areas in the brain and improve aphasia symptoms.^[33]^ This is also in line with somnolence’s principles of treatment; The Bahui acupoints can be used to treat diseases related to organs, qi, blood, tendons, and bone marrow. Somnolence can be caused by blood stasis blocking the orifices (the blockage of the qi and blood channels in the head by blood stasis), liver and gallbladder dampness and heat (the state in which the liver and gallbladder are excessively damp and overactive), brain and spirit loss (the loss of sufficient nourishment of consciousness in the brain), heart and kidney depletion,^[32]^ involving organs and blood. As the kidney is the source of marrow and the brain is the sea of marrow, somnolence is also related to marrow. Therefore, Bahui acupoints such as LR13(Zhangmen), BL17(Geshu), RN12(Zhongwan), and GB39(Xuanzhong) were used.

### 4.5. Association rule analysis of acupoint selection and compatibility

According to the analysis of association rules, the most frequently used core acupoint combinations in Table 4 are “KI3(Taixi) KI6(Zhaohai),” “KI4(Dazhong) HT5(Tongli),” “ST45(Lidui) SJ10(Tianjing),” “LI3(Sanjian) ST45(Lidui) SJ10(Tianjing),” “LI3(Sanjian) ST45(Lidui),” and “LI3(Sanjian) SJ10(Tianjing).” The last 4 combinations can be merged into the “LI3(Sanjian) ST45(Lidui) SJ10(Tianjing)” combination because they are closely related to ST45(Lidui), SJ10(Tianjing), and LI3(Sanjian). Both the KI3(Taixi) and KI6(Zhaohai) acupoints belong to the Foot Shaoyin Kidney Meridian’s acupoints. KI3(Taixi) is the Shu acupoint and yuan-primary acupoint of the Kidney Meridian, and KI6(Zhaohai) is the intersection acupoint of the Kidney Meridian and the Yinqiao Meridian. Somnolence was found in the “meridian diseases”(The name of a Traditional Chinese Medicine disease refers to the pathological changes in the organs that extend to the corresponding meridians, reflecting the disease pattern along the meridians) of the kidney meridian.^[16]^ As the yuan-primary acupoint, KI3(Taixi) has the effect of adjusting the deficiency and excess of its visceral meridians,^[17]^ and often plays a role in tonifying kidney qi(improving kidney function) in practical applications. The kidney meridian and Yinqiao meridian meet at the KI6(Zhaohai) acupoint, and Yinqiao meridian can control eyelid opening and closing. At the same time, somnolence is one of the diseases that KI6(Zhaohai) acupoint can be used for treatment. Therefore, acupuncture KI6(Zhaohai) can achieve the therapeutic effect of both kidney and Yinqiao meridians on somnolence. In addition, Zhaohai is commonly used in practical applications to tonify the kidneys and nourish yin. Therefore, the 2 acupoints were combined to play the role of tonify the kidneys. So it can be considered that this acupoint combination is suitable for the type of kidney essence deficiency with somnolence. KI4(Dazhong) is a Luo acupoint of the kidney meridian, which has a direct therapeutic effect on somnolence. In practical applications, it is often used for tonifying the kidney and absorbs qi. HT5(Tongli) is the acupoint of the heart meridian, and can be used to regulate mind and energy through the heart meridian. HT5(Tongli) is often used to calm and nourish the heart. Studies have indicated that acupuncture treatment mainly using HT5(Tongli) and KI4(Dazhong) can effectively improve cognitive function in dementia patients,^[34]^ which confirms the rationality of the combination of the 2 acupoints. ST45(Lidui) is the Jing acupoint of the stomach meridian, which is used to treat diseases such as heat syndrome, pain syndrome, and madness. LI3(Sanjian) is the Shu acupoint of the large intestine meridian, and is used to treat pain and heat syndromes. In addition, in the *A-B Classic of Acupuncture and Moxibustion*, it is also recorded that LI3(Sanjian) acupoint is mainly used to treat “excessive sleep,”^[19]^ which is equivalent to treating the somnolence. SJ10(Tianjing) is the He acupoint of the Sanjiao meridian, and is mainly used to treat deafness, pain, scrofula, epilepsy, and other syndromes. From a comprehensive perspective, the 3 acupoints (ST45(Lidui), LI3(Sanjian), SJ10(Tianjing)) are all yang meridian points, and they are mainly used to treat heat syndrome. When combined, they have the functions of clearing heat and relieving excess heat. The stomach, large intestine, and San jiao, they all belong to the category of the 6 fu organs, covering the upper, middle, and lower San jiao. The 3 acupoints are used together to play the role of clearing and relieving excess heat in San jiao (The visceral structure in Traditional Chinese Medicine refers to the 3 regions of the human body, namely the upper, middle, and lower parts). Somnolence is associated with excess heat syndrome of the liver and gallbladder dampness heat,^[32]^ and it can be considered that the 3 acupoints are combined for this syndrome type of somnolence.

Based on the analysis results of the association rules in Table 5, the acupoint combinations with strong association are similar to the highest frequency core acupoint combinations. Most of them are obtained by adding and subtracting the combinations of “KI3(Taixi) KI6(Zhaohai)” and “LI3(Sanjian) ST45(Lidui) SJ10(Tianjing)” in the above highest frequency acupoint combinations, corresponding to the treatment of 2 major types of somnolence: deficiency and excess, with 2 methods of supplementing deficiency and purging excess. Other acupoints with a lower frequency of occurrence are SJ8(Sanyangluo), BL18(Ganshu), DU20(Baihui), and LI2(Erjian). SJ8(Sanyangluo), BL18(Ganshu), and LI2(Erjian) tend to relieve excess, and can be used as acupoint combinations to strengthen the function of relieving excess, or as assistant acupoints for differentiation of meridians (It refers to determining acupoints by identifying the meridians to which a disease belongs.). The DU20(Baihui) acupoint belongs to the governor meridian, which is one of the meridians commonly responsible for somnolence. It also has the function of tonifying deficiencies and relieving excess. Regardless of the type of deficiency or excess, somnolence can be combined with this acupoint to achieve close local treatment.

### 4.6. Analysis of clustering results

As shown in Table 6, according to cluster analysis, 5 groups of effective cluster acupoint core combinations were obtained. Compared with the association analysis of core acupoint combinations, it can be seen that there are also relatively novel acupoint selection ideas for somnolence besides the addition and subtraction changes based on the association analysis of the 3 highest frequency acupoint combinations. Combination 1:LI2(Erjian) was added based on the core combination of “LI3(Sanjian) ST45(Lidui) SJ10(Tianjing)” and “KI3(Taixi) KI6(Zhaohai).” The location and main treatment of LI2(Erjian) were similar to those of LI3(Sanjian). In *A-B Classic of Acupuncture and Moxibustion*, “Excessive sleep” is the disease where 2 acupoints are used to treat at the same time, So these acupoints can play an important role in strengthening the treatment of excess heat syndrome. The combination of KI3(Taixi) and KI6(Zhaohai) acupoints, which take tonifying deficiency as the main function, is used with acupoints that can eliminate excess evil(A treatment method in Traditional Chinese Medicine that suppresses excessive hyperactivity of the body) at the same time. This choice is based on the consideration of the mixed effects of excess and deficiency in somnolence and the use of a treatment method that takes into account both the symptoms and the body; On the basis of “KI4(Dazhong) HT5(Tongli),” combination 2 adds 4 acupoints, BL17(Geshu), ST31(Biguan), LU1(Zhongfu), and LI13(Shouwuli). BL17(Geshu) is mainly used to treat spleen and stomach diseases such as vomiting and stomach pain, lung diseases such as cough and asthma and blood syndromes such as hematochezia and hematemesis. It can regulate the lung, spleen and stomach’s qi and blood. ST31(Biguan) is mainly used to treat asthenia flaccidity. It is related to the kidney and has the function of tonifying asthenia. LU1(Zhongfu), which is mainly used to treat cough, asthma, chest and back pain and other diseases, is the intersection acupoint of the Taiyin meridians of the hands and feet (Lung Meridian and Spleen Meridian), that is, the acupoint that can regulate both the lungs and spleen. LI13(Shouwuli) is mainly used to treat arm pain. As a acupoint of the large intestine meridian, it can regulate the function of the large intestine, and also mainly discharge excess (Suppress the excessive state of the human body). On the whole, this combination involves heart, lung, spleen, stomach, kidney, blood, large intestine and other human elements. On the basis of taking into account the treatment of deficiency and excess syndrome, the combination also takes into account the multiple connections between somnolence and the 5 zang organs and blood, it can be used to dredges the blood between the 5 zang organs, and this reflects the distinct concept of overall conditioning (considering the connections between organs throughout the body when diagnosing and treating diseases); The acupoint selection of combination 3 mainly involves the liver, spleen and kidney to exert the function of tonifying the 3 viscera, and the acupoint ST41(Jiexi) of the stomach meridian can be used to treat multiple syndromes of deficiency and excess. This acupoint is used here for its function of tonifying the spleen and stomach. According to this function, this combination is suitable for the deficiency syndrome of somnolence. Somnolence is often caused by emotional disturbance, which can easily damage the liver and spleen and consume the kidney essence. This group of acupoints takes into account the nature and nurture(Traditional Chinese Medicine regards the innate functions of life as nature, and the bodily substances obtained during the process of growth as nurture), and also pays attention to the liver injury and conditioning at the initial stage of the disease; The meaning of Combination 4 has been elaborated in association rule analysis; Combination 5 acupoint selection can be seen as a different acupoint selection for the deficiency type of somnolence, and its function is similar to that of Combination 3. Acupoint selection of Combination 5 was more streamlined. The acupoint RN12(Zhongwan), which is often used to regulate the function of the spleen and stomach, and the 3 acupoints RN6(Qihai), BL43(Gaohuang), ST36(Zusanli), which are mainly used to treat various deficiency disorders, were selected for the purpose of tonifying the nature and nurture.

The article also has some shortcomings. For example, the acupoint selection prescriptions collected from ancient medical books are mostly single acupoint prescriptions, and the number of compound prescriptions is small, which is not conducive to the analysis and research of more sufficient evidence. In another example, when combining the literature search of modern acupuncture and moxibustion in the treatment of somnolence, it was found that the number of studies on this disease was small, and sometimes it could not provide direct modern literature evidence support, which needs to be supported by indirect research literature on other diseases with somnolence as the manifestation. This not only lacks the most powerful modern evidence, but also reflects the lack of attention and research in this field. Of course, the advantages of this article are also evident. First of all, the discussion in this paper is mainly completed through the theory of Traditional Chinese Medicine, which is conducive to the expression of the original ideas of ancient physicians, and also helps readers to deepen their understanding of acupuncture and moxibustion theory and even Chinese Medicine culture. Secondly, the data in this article are all sourced from the *Encyclopaedia of Traditional Chinese Medicine*, which collects a large number of ancient Chinese medical books. The content is consistent with the original content of the books and will not deviate from the original viewpoints of the books. At the same time, it is convenient for researchers and readers to access ancient medical books. Thirdly, this paper explores a new method to treat somnolence from the perspective of acupuncture and moxibustion, which is less studied at present and has certain innovation, and can inspire relevant researchers to consider the treatment of this disease from the perspective of Traditional Chinese Medicine.

In summary, acupuncture and moxibustion in ancient Chinese medical books mostly used distal acupoints. The kidney meridian, large intestine meridian, bladder meridian, Sanjiao meridian, governor meridian’s acupoints are often used, and Shu acupoints, Luo acupoints, Bahui acupoints on specific acupoints are often used. Acupuncture prescriptions are mostly from the perspective of tonifying deficiency, discharging excess and considering both deficiency and excess. Core acupoint combinations such as “KI3(Taixi) KI6(Zhaohai),” “KI4(Dazhong) HT5(Tongli),” “LI3(Sanjian) ST45(Lidui) SJ10(Tianjing)” are often used, and acupoints are selected according to syndrome differentiation and overall regulation. This is different from the idea and selection of acupoints of the heart and gallbladder meridians in the standard of modern acupuncture and moxibustion for the treatment of somnolence,^[32]^ but there are also similarities. On the one hand, with the development of science and technology, modern medical workers have better conditions to carry out clinical scientific research activities. The acupoints of the heart and gallbladder meridians have shown good curative effects on somnolence in clinical efficacy experiments.^[35]^ On the other hand, although there are some differences in the main acupoints, ancient and modern prescriptions have both selected DU20(Baihui), ST36(Zusanli), and SP6(Sanyinjiao), and the treatment idea with regulating mind as the core is also the same. In modern clinical practice, while using the effective acupoints confirmed by experiments, we should also pay attention to the inheritance of the concept of overall regulation in ancient medical books, and pay attention to the combination of indications and curative effects of acupoints and meridians, simplify acupoints while taking into account disease-related visceral conditions, so as to achieve the best curative effect on somnolence. As a common disease affecting people’s health, more literature and clinical research on somnolence should be conducted in the future. The acupuncture and moxibustion therapy, which has been mentioned many times in ancient medical books for the treatment of somnolence, should still be studied more to fully prove its effectiveness. When modern medical technology encounters difficulties in treating somnolence and other diseases, it may be worth considering viewing and treating them from the perspective of Traditional Chinese Medicine. This may not only discovers better treatment methods, but also provides new breakthroughs for the development and innovation of medicine.

## Author contributions

**Conceptualization:** Hai-qing Zeng

**Data curation:** Hai-qing Zeng, Ze-dong-fang Yuan, Run-tian Wu, A-ling Tang.

**Formal analysis:** Hai-qing Zeng, Ze-dong-fang Yuan, Run-tian Wu, A-ling Tang.

**Methodology:** Hai-qing Zeng.

**Software:** Hai-qing Zeng.

**Supervision:** Hai-qing Zeng, A-ling Tang, Zhi-sheng Huang.

**Validation:** Hai-qing Zeng, Ze-dong-fang Yuan, A-ling Tang, Zhi-sheng Huang.

**Visualization:** Hai-qing Zeng, Ze-dong-fang Yuan.

**Writing – original draft:** Hai-qing Zeng, Ze-dong-fang Yuan, Run-tian Wu, A-ling Tang.

**Writing – review & editing:** Hai-qing Zeng, Ze-dong-fang Yuan, Run-tian Wu, A-ling Tang, Zhi-sheng Huang.

## Supplementary Material


