# Supplementary material for: Analysis of acupoint selection rules of acupuncture and moxibustion in ancient medical books for the treatment of somnolence: A review
Source: Medicine (Baltimore). 2025 Mar 14;104(11):e41676. doi: 10.1097/MD.0000000000041676 (PMC11922414; doi:10.1097/MD.0000000000041676)
Supplement: Supplementary file 1 [file medi-104-e41676-s001.pdf]

· 文献研究 ·

基于中医传承辅助平台挖掘针灸治疗膝骨关节炎的取穴规律

邹媛[1] 朱翔宇[1] 陈亚飞[2] 卫博文[2] 刘志丹[1] 李巧巧[1] 陈媛琳[1] 黄怡然[1] 李文迅[1]

【摘要】 **目的** 通过中医传承辅助平台（TCMISS V2.5），对现有且经过临床检验的针灸治疗膝骨关节炎（Knee Osteoarthritis，KOA）处方进行数据统计，从而总结针灸配伍规律，并挖掘新的组穴处方。**方法** 计算机检索建库至 2020 年 12 月 CNKI 期刊数据库中关于针灸治疗 KOA 的临床研究文献，严格按照纳入和排除标准，筛选针灸穴位处方并录入 TCMISS，运用软件进一步分析，集成数据规律。**结果** 共纳入 144 首穴位处方，包含 11 条经脉，46 个穴位；最常使用的穴位有犊鼻（12.7%）、内膝眼（12.7%）、阳陵泉（11.0%）、血海（10.3%）、梁丘（9.8%）、足三里（9.6%）、阴陵泉（8.2%）等；以足阳明胃经（42.3%）穴位出现的频率最高，其次是足太阴脾经（26.7%）、足少阳胆经（18.9%）、足太阳膀胱经（7.1%）、足厥阴肝经（1.6%）；出现不同穴性 14 种，共计 714 次，其中以合穴（42.4%）出现的频次最高，其次是下合穴（30.7%）、郄穴（13.2%）、交会穴（3.9%）、背俞穴（2.5%）、八会穴（2.4%）、原穴（1.5%）；位于前 6 的穴位组合为“内膝眼－犊鼻”“内膝眼－阳陵泉”“犊鼻－阳陵泉”“内膝眼－血海”“犊鼻－血海”“血海－梁丘”；得到“阳陵泉，足三里，大肠俞，血海”“合谷，内膝眼，风市，委阳，犊鼻”“梁丘，阳陵泉，阴陵泉，大肠俞，肾俞”3 组备选新方。**结论** 针灸治疗 KOA 重视腧穴的近治作用，多使用局部取穴；归经以胃经、脾经、胆经、膀胱经为主；常配伍使用合穴、下合穴、八会穴、背俞穴；重视脏腑整体，调元气以通经络。

【关键词】 膝骨关节炎；针灸；中医传承辅助平台；组穴规律；数据挖掘

【中图分类号】R684.3 **【文献标识码】**A

Analysis of Acupoint Selection Rules of Acupuncture and Moxibustion Treatment of Knee Osteoarthritis Based on TCMISS

ZOU Yuan[1]，ZHU Xiang－yu[1]，CHEN Ya－fei[2]，WEI Bo－wen[2]，LIU Zhi－dan[1]，LI Qiao－qiao[1]，CHEN Yuan－lin[1]，HUANG Yi－ran[1]，LI Wen－xun[1]

（1. School of Acupuncture and Tuina，Beijing University of Chinese Medicine，Beijing 100029；2. School of Traditional Chinese Medicine，Beijing University of Chinese Medicine，Beijing 100029）

【Abstract】 **Objective** Through the TCMISS V2.5，statistics on existing and clinically tested prescriptions for acupuncture treatment of knee osteoarthritis（KOA），so as to summarize the rules and dig new acupoint prescriptions. **Methods** The clinical research literature on acupuncture and moxibustion treatment of KOA in the CNKI from 1992 to 2020 was searched. According to the inclusion and exclusion conditions，acupuncture point prescriptions were selected and entered into TCMIS for further analysis and mining rules. **Results** A total of 144 acupoint prescriptions were included，including 11 meridians and 46 acupoints. The most commonly used acupoints were Dubi（ST35）（12.7%），Neixiyan（EX－LE4）（12.7%），Yanglingquan（GB34）（11.0%），Xuehai（SP10）（10.3%），Liangqiu（ST34）（9.8%），Zusanli（ST36）（9.6%），Yinlingquan（SP9）（8.2%）. The frequency of acupuncture points in the Stomach Channel of Foot－Yangming（42.3%）is highest，followed by Spleen Channel of Foot－Taiyin（26.7%），Gallbladder Channel of Foot－Shaoyang（18.9%），Urinary Bladder Channel of Foot－Taiyang（7.1%），Liver Channel of Foot－Jueyin（1.6%）. There are 14 different acupoint types，a total of 714 times，of which the He acupoint（42.4%）has the highest frequency，followed by Lower Xiahe acupoint（30.7%），Xi acupoint（13.2%），Jiaohui acupoint（3.9%），Beishu acupoint（2.5%），Bahui acupoint（2.4%），Yuan acupoint（1.5%）. The first 6 acupoint combinations are “Neixiyan（EX－LE4）－Dubi（ST35）”，“Neixiyan（EX－LE4）－Yanglingquan（GB34）”，“Dubi（ST35）－Yanglingquan（GB34）”，“Neixiyan（EX－LE4）－Xuehai（SP10）”，“Dubi（ST35）－Xuehai（SP10）”，“Xuehai（SP10）－Liangqiu（ST34）”；and“Yanglingquan（GB34）－Zusanli（ST36）－Dachangshu（BL25）－Xuehai（SP10）”，“Hegu（LI4）－Neixiyan（EX－LE4）－Fengshi（GB31）－Weiyang（BL39）－Dubi（ST35）”，“Liangqiu（ST34）－Yanglingquan（GB34）－Yinlingquan（SP9）－Dachangshu（BL25）－Shenshu

---

DOI：10.13935/j.cnki.sjzx.210806

基金项目：北京中医药大学重点攻关项目（2020－JYB－ZDGG－066）；北京中医药大学在读研究生项目（2018－JYB22－XS117）

作者单位：1. 北京中医药大学针灸推拿学院，北京 100029；2. 北京中医药大学中医学院，北京 100029

通信作者：李文迅，Email：agoodfriend@126.com

(C)1994-2022 China Academic Journal Electronic Publishing House. All rights reserved. http://www.cnki.net

(BL23)" 3 groups of new prescriptions. **Conclusion** Acupuncture and moxibustion treatment of KOA pays more attention to the recent treatment of acupoints, and uses local acupoint selection; the main meridians are stomach meridian, spleen meridian, gall bladder meridian, and bladder meridian; He acupoints, Xiahe acupoints, Bahui acupoints, and Backshu are often used in combination Points. The principle of treatment pay attention to the whole viscera, adjust vitality to clear the meridians.

【**Keywords**】 Knee Osteoarthritis; Acupuncture And Moxibustion; TCMISS; Rules of Acupoint Grouping; Data Mining

膝骨关节炎(Knee Osteoarthritis, KOA)以膝关节疼痛、肿大畸形、活动受限、骨摩擦音或伴有关节腔积液为主要临床表现,是一种复杂的周围性关节疾病[1],是老年人活动受限和身体残疾的主要原因之一[2],随人口老龄化进程日益严重[3]。研究表明,我国 40 岁以上中老年人 KOA 总患病率为 17.0%,其中男性 12.3%,女性 22.2%[4];在运动员中,KOA 患病率达到 30.0%[5];同时大约有 2400 万日本人[6],2700 万美国人受其影响和困扰[7]。KOA 治疗多采用减轻体重、运动疗法、物理康复、口服药物、关节腔注射和手术置换关节等方法[8]。近些年,临床关于中医治疗 KOA 的有效报道越来越多,针灸作为中医外治法的一种,在治疗 KOA 具有明确的镇痛效果、能扩大关节活动度,可有效降低缓解症状,且针刺具有安全、微创、简便、经济等优势,使之成为 KOA 患者有吸引力的治疗选择。现通过 TCMISS 对 CNKI 收录的针灸治疗 KOA 的相关期刊进行针灸穴位处方的分析,以期为临床工作者提供有价值的参考和理论依据。

1 资料与方法

1.1 文献来源与检索策略

以 CNKI 数据库为数据来源,以"针灸""针刺""针""膝关节骨性关节炎""膝骨关节炎""增生性膝骨关节炎""膝关节骨关节病""退行性膝关节炎""膝关节退行性病变""KOA"为检索词,时间设定为建库至 2020 年 12 月,得到 CNKI 数据库有关针灸治疗 KOA 的文献,按照纳入和排除标准筛选文献,确定最终纳入分析的文献。

1.2 纳入标准

①临床确诊为膝骨关节炎;②选穴组方明确,临床疗效判定为有效、显效、治愈;③腧穴选取来自十四经穴和经外奇穴的穴位;④治疗组采用针灸疗法,或以针灸疗法为主合并其他治疗方式。

1.3 排除标准

①使用非十四经穴及非经外奇穴疗法(如董氏奇穴、靳三针、全息针法、浮针等)的文献;②综述类、Meta 分析等数据挖掘类、理论探讨类、个案报道类、动物实验类等文献;③针灸处方不明确的文献;④无对照组的文献。

1.4 数据录入

依据上述纳入标准与排除标准,筛选出的最终用于录入分析的文献,将这些文献中治疗组出现的所有针灸处方录入 TCMISS,建立对应数据库。数据由 2 人核对录入。

1.5 术语规范

纳入针灸处方中涉及的所有穴位,参考国家标准《腧穴名称与定位》[9]进行统一规范。其中,压痛点取穴,统一归类为阿是穴;部分特殊穴位名称,需提供可靠的定位标准,若非主穴,则不计入本次研究。

1.6 数据分析

录入全部数据后,打开 TCMISS,分别通过"统计报表"和"数据分析"模块,进行使用穴位的频次统计、组方规律以及潜在新方的分析,并将所得结果导出保存。

2 结 果

2.1 一般情况

通过文献高级检索,初步获得符合条件的文献 297 篇,经仔细阅读筛选,最终纳入治疗处方 144 首,涉及穴位计 46 个。

2.2 穴位频次统计

144 首处方中,所有穴位共出现 955 次,十四正经穴位为 744 次,经外奇穴为 168 次,阿是穴为 43 次,频次 >10 的有 13 个穴位。其中犊鼻(12.7%)和内膝眼(12.7%)出现的频率最高,且成对出现。随后是阳陵泉(11.0%)、血海(10.3%)、梁丘(9.8%)、足三里(9.6%)、阴陵泉(8.2%)。结果见表 1。

表 1 穴位频次表(>10)

序号	穴位名称	频次(次)	频率(%)
1	犊鼻	121	12.7
2	内膝眼	121	12.7
3	阳陵泉	105	11.0
4	血海	98	10.3
5	梁丘	94	9.8
6	足三里	92	9.6
7	阴陵泉	78	8.2
8	鹤顶	47	4.9
9	阿是穴	43	4.5
10	三阴交	21	2.2
11	委中	17	1.8
12	膝阳关	16	1.7
13	悬钟	12	1.3

(C)1994-2022 China Academic Journal Electronic Publishing House. All rights reserved. http://www.cnki.net

2.3　穴位归经统计

共涉及 11 条经脉，其中阳经有 5 条、阴经有 6 条。以足阳明胃经（42.3%）穴位出现的频率最高，其次是足太阴脾经（26.7%）、足少阳胆经（18.9%）、足太阳膀胱经（7.1%）、足厥阴肝经（1.6%）。结果见表 2。

表 2　归经频次表

序号	穴位归经	个数	频次（次）	频率（%）
1	足阳明胃经	8	314	42.3
2	足太阴脾经	4	198	26.7
3	足少阳胆经	5	140	18.9
4	足太阳膀胱经	11	53	7.1
5	足厥阴肝经	3	12	1.6
6	任脉	3	9	1.2
7	足少阴肾经	2	6	0.8
8	手阳明大肠经	2	4	0.5
9	督脉	3	3	0.4
10	手厥阴心包经	1	2	0.3
11	手少阴心经	1	1	0.1

2.4　穴位的穴性情况

出现不同穴性 14 种（表 3），共计 714 次，无穴性穴位（包括经外奇穴 168 次和阿是穴 43 次）计 490 次。其中以合穴（42.4%）出现的频次最高，其次是下合穴（30.7%）、郄穴（13.2%）、交会穴（3.9%）、背俞穴（2.5%）、八会穴（2.4%）、原穴（1.5%）。

表 3　穴性频次表

序号	穴性	个数	频次（次）	频率（%）
1	合穴	7	303	42.4
2	下合穴	4	219	30.7
3	郄穴	1	94	13.2
4	交会穴	4	28	3.9
5	背俞穴	3	18	2.5
6	八会穴	3	17	2.4
7	原穴	4	11	1.5
8	输穴	2	7	1.0
9	募穴	1	5	0.7
10	络穴	2	4	0.6
11	经穴	2	4	0.6
12	八脉交会穴	1	2	0.3
13	井穴	1	1	0.1
14	荥穴	1	1	0.1

2.5　基于关联规则的组穴规律分析

通过 TCMISS“组方规律”分析模块实现。将支持度个数设置为 30（表明该穴位组合至少同时出现在 30 个处方中），置信度设为 0.9（表明该关联规则的可信程度为 90% 以上），则共有 157 条穴位组合，147 条关联规则，包含 9 个穴位，见图 1；将支持度个数设置为 60、置信度为 0.9，则共有 44 条穴位组合，27 条关联规则，包含 7 个穴位，见图 2。综合 2 次设置得到的信息，根据频次排序，对前 30 位的核心穴位组合（见表 4）和前 20 位的关联规则（见表 5）展示如下。其中，位于前 6 的穴位组合为“内膝眼－犊鼻”“内膝眼－阳陵泉”“犊鼻－阳陵泉”“内膝眼－血海”“犊鼻－血海”“血海－梁丘”，置信度最高的前 4 位为“梁丘，阴陵泉→阳陵泉”“血海，梁丘，阴陵泉→阳陵泉”“犊鼻，梁丘，阴陵泉→阳陵泉”“内膝眼，梁丘，阴陵泉→阳陵泉”。

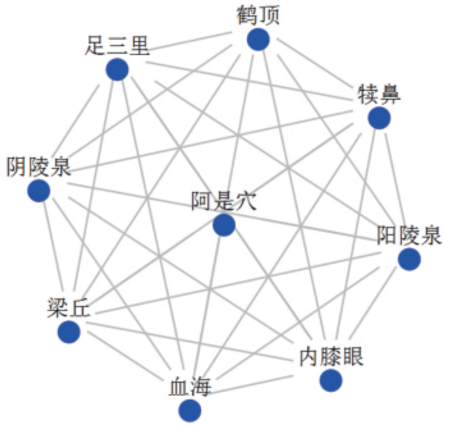

图 1　组穴模式 1

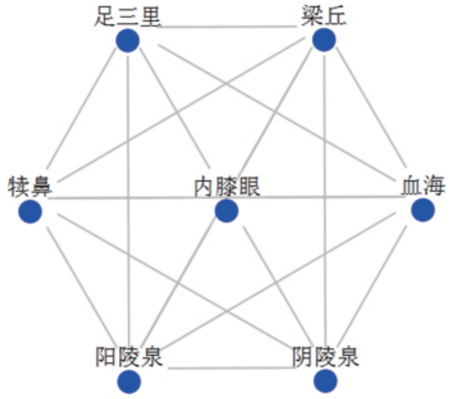

图 2　组穴模式 2

表 4　针灸治疗 KOA 的核心穴位组合

序号	穴位组合	频次（次）	序号	穴位组合	频次（次）
1	内膝眼－犊鼻	111	16	足三里－阳陵泉	74
2	内膝眼－阳陵泉	91	17	内膝眼－犊鼻－梁丘	74
3	犊鼻－阳陵泉	87	18	内膝眼－犊鼻－足三里	71
4	内膝眼－血海	84	19	犊鼻－血海－梁丘	71
5	犊鼻－血海	83	20	血海－足三里	70
6	血海－梁丘	82	21	内膝眼－血海－梁丘	70
7	内膝眼－犊鼻－阳陵泉	82	22	血海－梁丘－阳陵泉	70
8	梁丘－阳陵泉	81	23	内膝眼－阴陵泉	69
9	犊鼻－梁丘	80	24	犊鼻－阴陵泉	68
10	内膝眼－梁丘	79	25	内膝眼－血海－阳陵泉	68
11	内膝眼－足三里	79	26	内膝眼－梁丘－阳陵泉	68
12	血海－阳陵泉	79	27	犊鼻－梁丘－阳陵泉	68
13	内膝眼－犊鼻－血海	78	28	内膝眼－犊鼻－血海－梁丘	67
14	犊鼻－足三里	77	29	内膝眼－阳陵泉－阴陵泉	66
15	阳陵泉－阴陵泉	75	30	犊鼻－血海－阳陵泉	66

(C)1994-2022 China Academic Journal Electronic Publishing House. All rights reserved.　http://www.cnki.net

表5 针灸治疗KOA穴位组合的关联规则

序号	规则	置信度
1	梁丘,阴陵泉→阳陵泉	0.983 606 557
2	血海,梁丘,阴陵泉→阳陵泉	0.982 142 857
3	犊鼻,梁丘,阴陵泉→阳陵泉	0.981 132 075
4	内膝眼,梁丘,阴陵泉→阳陵泉	0.980 769 231
5	内膝眼,犊鼻,梁丘,阴陵泉→阳陵泉	0.979 591 837
6	内膝眼,血海,梁丘,阴陵泉→阳陵泉	0.979 591 837
7	犊鼻,血海,梁丘,阴陵泉→阳陵泉	0.979 591 837
8	梁丘,足三里,阴陵泉→阳陵泉	0.978 260 870
9	内膝眼,犊鼻,血海,梁丘,阴陵泉→阳陵泉	0.978 260 870
10	血海,梁丘,足三里,阴陵泉→阳陵泉	0.976 190 476
11	犊鼻,梁丘,足三里,阴陵泉→阳陵泉	0.975 609 756
12	内膝眼,梁丘,足三里,阴陵泉→阳陵泉	0.974 358 974
13	内膝眼,阿是穴→犊鼻	0.973 684 211
14	内膝眼,犊鼻,梁丘,足三里,阴陵泉→阳陵泉	0.972 972 973
15	犊鼻,血海,梁丘,足三里,阴陵泉→阳陵泉	0.972 972 973
16	内膝眼,血海,梁丘,足三里,阴陵泉→阳陵泉	0.972 222 222
17	内膝眼,犊鼻,血海,梁丘,足三里,阴陵泉→阳陵泉	0.970 588 235
18	血海,阴陵泉→阳陵泉	0.967 741 935
19	内膝眼,血海,阴陵泉→阳陵泉	0.963 636 364
20	犊鼻,血海,阴陵泉→阳陵泉	0.962 264 151

2.6 基于熵聚类的新核心穴位组合挖掘及规律分析

通过TCMISS“新方分析”模块实现。设置相关系数为8、惩罚度为2[10],得到32组2穴组合、18组3穴组合、5组4穴组合,通过系统熵聚类,演算出3对穴位组合,得到“阳陵泉,足三里,大肠俞,血海”“合谷,内膝眼,风市,委阳,犊鼻”“梁丘,阳陵泉,阴陵泉,大肠俞,肾俞”3组备选新方组合。结果见表6。

3 讨 论

膝骨关节炎,属于中医学“痹症”“骨痹”范畴,病位在膝部筋骨,属本虚标实之证,基本病机是气血瘀滞,筋骨失养[11]。以整体观辨证论治,正气不足是发病的内在病因,外感风、寒、湿、热之邪是为其外因。邪气入侵,积聚在膝关节,阻滞气血经络的正常运行,导致疼痛和局部活动受限,而这两个症状是互相关联的[12],由于疼痛不敢动而加强活动受限,不活动又导致瘀滞,使经络进一步闭阻,局部粘连加重,从而疼痛也更加剧烈。肾主骨生髓,骨关节病多与肾有关,老年人多肝肾亏虚、身体各方面机能减退,气血不能濡养,经脉血管硬化,肌肉萎缩肌力下降。这也是本病在中老年人群多发的原因之一。故KOA治疗上应以局部疏通为要,兼以整体补益,通经活络,壮骨止痛,运行气血。

根据表1可知,最常使用的穴位有犊鼻、内膝眼、阳陵泉、血海、梁丘、足三里、阴陵泉等,是以膝关节局部取穴为主,“腧穴所在,主治所在”,发挥腧穴的近治作用,针对性治疗膝关节骨性关节炎。其中,犊鼻与内膝眼、阳陵泉与阴陵泉、血海与梁丘,这三对穴组常做透刺法,或加电针相连,以促进疗效。犊鼻是足阳明胃经腧穴,“是主血所生病者……膝膑肿痛”,内膝眼是经外奇穴,此二穴常用治膝部肿痛、屈伸不利以及下肢痿痹,位于髌韧带两侧凹陷处,是KOA常见的压痛点[13]。阳陵泉是足少阳胆经合穴、胆下合穴,且是八会穴之筋会,是治疗筋脉麻痹之要穴,对痉证、痿证、痹证等有较好的治疗作用[14]。阴陵泉是足太阴脾经合穴,可健脾除湿化滞,与阳陵泉同用,共奏舒筋活络、祛风除湿、通痹止痛之功。血海又名血郄,为足太阴脾经脉气所发,脾主统血,温五脏,为气血归聚之海,针刺血海可活血和营,疏经通络[15]。梁丘为足阳明胃经之郄穴,乃经气深聚之穴,有调和气血之功。《千金要方》记载“梁丘、曲泉、阳关主筋挛,膝不得屈伸,不可以行[16]。”足三里是足阳明胃经合穴、胃下合穴,有“人体第一长寿穴”之称,具有调和气血、养胃健脾、强身健体的作用,意在以后天养先天。七穴相配,祛除风寒湿邪,补益正气,标本兼顾治疗KOA。

“膝者,筋之府也”,足六经皆从膝循行经过,循经取穴是针灸选穴的基本原则,“经脉所过,主治所及”,故足六经皆可治疗膝关节病症。从人体位置分布来看,膀胱经位于背面,胃经位于正面,少阳经位于外侧面,脾经位于内侧面,全方位治疗膝关节。表2显示本次纳入的针灸处方中,以足阳明胃经穴位出现的频率最高,《内经》云“治痿独取阳明”“阳明者,五脏六腑之海,主润宗筋,宗筋主束骨而利机关[17]。”此亦可运用于痹症,一可增加气血濡养,二可疏通局部阻滞,通利关节。穴位使用个数上最多的经脉是足太阳膀胱经,太阳为一身之藩篱,主卫外之功,外邪入侵太阳首当其冲。另外,背俞穴主要集中在膀胱经,内应脏腑,滋养全身。足少阳胆经频次和穴位个数都排在第3位,足少阳胆经是枢经之一,是人体经脉的关键所在,可使枢机流畅,枢

表6 针灸治疗KOA的新方挖掘

序号	穴位组合1	穴位组合2	备选新方
1	阳陵泉,足三里,大肠俞	阳陵泉,足三里,血海	阳陵泉,足三里,大肠俞,血海
2	合谷,内膝眼,犊鼻	内膝眼,风市,委阳,犊鼻	合谷,内膝眼,风市,委阳,犊鼻
3	梁丘,阳陵泉,阴陵泉,大肠俞	梁丘,阳陵泉,阴陵泉,肾俞	梁丘,阳陵泉,阴陵泉,大肠俞,肾俞

(C)1994-2022 China Academic Journal Electronic Publishing House. All rights reserved. http://www.cnki.net

经畅则诸经通，经脉通则气血行，气血行则人体生理功能正常[18]。“太阳为开，阳明为阖，少阳为枢”，太阳常多血少气，少阳常多气少血，阳明常多气多血，三阳相配，气血相通，促进KOA的治疗[19]。足太阴脾经主脾络胃，脾胃为后天之本，吸收水谷精微，使气血生化有源，并将津液气血输布全身，营养四肢百骸，使之发挥正常机能。屈伸不利多责之与筋，肝在体合筋，“肝气衰则筋不能动，肝气实则筋脉拘挛[20]。”肾主骨生髓，取足厥阴肝经和足少阴肾经，肝肾同治，强筋壮骨。任脉和督脉，一为阴脉之海，一为阳脉之海，共同调节阴阳气血。

由表3可知，合穴在频次和穴位个数上都是最多的。足六经中除足少阴肾经，其他五经的合穴皆包含在本研究之中。合穴是五输穴之一，“所入为合”，意为脉气至此犹水汇入海，最为盛大，是经气由此深入，进而会合于脏腑的部位，可固本培元，调动脏腑元气以攻局部病灶，一般位于肘膝关节附近，可发挥腧穴近治作用。在频次和穴位个数上居第2的是下合穴，研究中包含足三里、阳陵泉、委中、委阳4个，位于足三阳经膝关节及以下部位，同样具有近治作用，且可借助六腑之气以通经络。本次研究中用于治疗KOA的八会穴有4个，骨会大杼、髓会悬钟、筋会阳陵泉、血会膈俞，骨髓筋血与KOA息息相关，紧密结合，针对性治疗。骨之气，皆会于大杼，“主膝痛不可屈伸”，《难经本义·四十五难》提到“骨者髓所养，髓自脑下注于大杼……渗注骨节。”大杼对督脉、对阳经经气的乃至全身筋骨系统的调节都有重要作用[21]；《千金要方·卷三十针灸下》载“悬钟主湿痹流肿，髀筋急瘈，胫痛……[22]”针刺悬钟有益髓壮骨的功效[23]；膈俞有止血、活血、补血、调血之功效，是治血证之要穴，此处用治KOA，本着“治风先治血，血行风自灭”的理论基础，有利于祛除风邪，补血养筋。背俞穴用了膈俞、大肠俞、肾俞、脾俞4个，有补益脏腑功能，增强整体的作用，同时带动对局部病症的治疗效果，配伍合穴、下合穴使用，促进脏腑经气灌注，从而提高疗效。穴性规律分析中还包含了十二原穴中足少阴肾经、手阳明大肠经、足厥阴肝经、足少阳胆经4经的原穴，阳明气血旺盛，大肠与胃常关联在一起，促进饮食物的消化吸收。

图1、图2是针刺治疗KOA常见的两种穴位组合。其中有一些穴组，常成对出现，以“犊鼻-内膝眼”为典型，其次有“阴陵泉-阳陵泉”“血海-梁丘”。研究发现[24]，深刺犊鼻、内膝眼二穴对KOA有较快止痛效果。阳陵泉和阴陵泉，两穴一阴一阳，阴阳互助，扶正祛邪，镇痛却疾[25]。针刺血海、梁丘能使通调气血，使膝关节周围肌肉动力平衡，促进关节功能恢复正常[26]。关联规则提示前面穴位出现的情况下，后者出现的可能性，结合图1、图2，说明运用针灸治疗KOA，很大概率下犊鼻、内膝眼、阴陵泉、阳陵泉、血海、梁丘会同时使用。表4的核心穴位组合也主要由这6个穴位以及足三里穴构成。说明这种穴位组合是经受住临床检验的，疗效是确切可靠的。TCMISS系统熵聚类的结果提示我们临床或可将“阳陵泉，足三里，大肠俞，血海”“合谷，内膝眼，风市，委阳，犊鼻”“梁丘，阳陵泉，阴陵泉，大肠俞，肾俞”3组穴组纳入针灸治疗KOA的配伍备选方案。此处出现了大肠俞、肾俞、委阳、合谷、风市5个非高频使用穴位，前3个属足太阳膀胱经，且大肠俞、肾俞是背俞穴，补益脾肾，肾虚痹可配伍；委阳是三焦之下合穴，通运三焦津液气机，着痹可配伍；合谷是手阳明大肠经原穴，针刺合谷穴可影响内源性阿啡肽[27]，发挥镇痛作用，痛痹可配伍；风市属足少阳胆经，治风要穴，行痹可配伍。

本研究应用TCMISS对针灸治疗KOA取穴规律进行探索浅析，发现针灸治疗KOA重视腧穴的近治作用，多使用局部取穴；归经以胃经、脾经、胆经、膀胱经为主；常配伍使用合穴、下合穴、八会穴、背俞穴；重视脏腑整体，调元气以通经络。本研究得到的穴位频次结果、组穴规律、备选新方，可为临床针灸治疗KOA提供参考，稳定的核心穴组可在无复杂辨证条件下推广使用，获得的新穴组可根据临床辨证选择性配伍使用。但本研究仍存在一定不足，一些非使用十四正经穴位的特殊针法未能纳入研究，挖掘层次尚且较浅，后续可从文献学角度试寻针灸治疗KOA主要取穴的溯源与使用变迁、特殊针法与传统针法的关联等。

参考文献

[1] HUSSAIN S M，NEILLY D W，BALIGA S，et al. Knee osteoarthritis: a review of management options [J]. Scott Med J，2016，61(1)：7-16.

[2] CHEN P，GAO L，SHI X，et al. Fully automatic knee osteoarthritis severity grading using deep neural networks with a novel ordinal loss [J]. Comput Med Imaging Graph，2019(75)：84-92.

[3] KAN H S，CHAN P K，CHIU K Y，et al. Non-surgical treatment of knee osteoarthritis [J]. Hong Kong Med J，2019，25(2)：127-133.

[4] 帖小佳，郑如庚，赵梦，等. 中国中老年人膝关节骨关节炎患病率的Meta分析[J]. 中国组织工程研究，2018，22(4)：650-656.

[5] MADALENO F O，SANTOS B A，ARAUJO V L，et al. Prevalence of knee osteoarthritis in former athletes:

(下转第1420页)

(C)1994-2022 China Academic Journal Electronic Publishing House. All rights reserved. http://www.cnki.net

phase defect cycles and outcome of progesterone treatment in patients with recurrent spontaneous abortion [J]. Am J Obstet Gynecol, 1988, 158(2): 225 – 232

[4] HIGGINS JPT, GREEN S, THE COCHRANE COLLABORATION. Cochrane Handbook for Systematic Reviews of Interventions [EB/OL]. Version 5. 0. 2. 2009. available at: http: //www. cochrane – handbook. org.

[5] 陈玮文. 110 例复发性流产患者的临床治疗与观察 [J]. 现代诊断与治疗, 2017, 28(14): 2634 – 2635.

[6] 饶育梅. 补肾安胎方联合黄体酮治疗复发性流产 39 例 [J]. 中国药业, 2014, 23(22): 112 – 114.

[7] 韩春艳, 孙自学, 宋艳丽. 补肾安胎饮对肾虚型不明原因复发性流产患者的临床疗效 [J]. 中成药, 2019, 41(12): 3067 – 3071.

[8] 党慧敏, 刘艳巧, 刘润侠, 等. 补肾活血方联合地屈孕酮对复发性流产患者临床疗效及 Th1/Th2 型细胞因子的影响 [J]. 西安交通大学学报(医学版), 2014, 35(6): 832 – 836.

[9] 钱艳清, 马大正, 孙云. 补肾疏肝方治疗肾虚肝郁型复发性流产的临床效果 [J]. 中国医药导报, 2017, 14(16): 86 – 89.

[10] 华乐荣. 黄体酮胶囊治疗复发性自然流产疗效观察 [J]. 中国实用医刊, 2014, 41(1): 113 – 114.

[11] 何冬华. 寿胎丸合四物汤加减联合黄体酮治疗复发性流产肾虚血瘀型疗效观察 [J]. 实用中医药杂志, 2018, 34(4): 458 – 459.

[12] 刘筱茂. 胎宝汤治疗脾肾阳虚型滑胎临床研究 [J]. 陕西中医, 2016, 37(11): 1506 – 1507.

[13] 许春艳, 孙晶. 泰山磐石散加减治疗复发性流产患者 60 例临床观察 [J]. 世界中西医结合杂志, 2017, 12(3): 369 – 371.

[14] 付静. 益肾健脾祛瘀方联合黄体酮治疗早期复发性流产临床观察 [J]. 光明中医, 2019, 34(8): 1260 – 1262.

[15] 李顺景. 茵陈术附汤联合黄体酮治疗脾肾阳虚复发性流产随机平行对照研究 [J]. 实用中医内科杂志, 2016, 30(10): 56 – 58.

[16] 赵敏霞, 赵新敏. 中西医结合治疗先兆流产习惯性流产患者临床疗效以及对孕酮及 β – HCG 水平的影响 [J]. 临床心身疾病杂志, 2017, 23(5): 110 – 111, 114.

[17] 任建荣, 张文静, 范艳霏. 自拟复黄保胎方对黄体功能不全型复发性流产患者内分泌功能的影响 [J]. 现代中西医结合杂志, 2016, 25(14): 1548 – 1551.

[18] 代玉红, 庞秀香. 自拟中药联合黄体酮对不明原因复发性自然流产患者影响的研究 [J]. 系统医学, 2017, 2(17): 30 – 34.

(收稿日期: 2021 – 03 – 23)

(上接第 1410 页)

a systematic review with meta – analysis. [J]. Braz J Phys Ther, 2018, 22(6): 437 – 451.

[6] NAGAO M, ISHIJIMA M, KANEKO H, et al. Physical activity for knee osteoarthritis [J]. Clin calcium, 2017, 27(1): 25 – 30.

[7] GWAM C U, ETCHESON J I, GEORGE N E, et al. Presentation of Knee Osteoarthritis in the Emergency Department: A Problem Worth Mentioning? [J]. Surg Technol Int, 2017(31): 277 – 284.

[8] VITALONI M, BOTTO – VAN BEMDEN A, ROSA MAYA SCIORTINO CONTRERAS, et al. Global management of patients with knee osteoarthritis begins with quality of life assessment: a systematic review [J]. BMC Musculoskelet Disord, 2019, 20(1): 493.

[9] 中华人民共和国国家质量检验检疫总局. GB/T 12346 – 2006 腧穴名称与定位 [S]. 北京: 中国标准出版社, 2006: 7 – 43.

[10] 杨洪军, 唐仕欢, 卢鹏. TCMISS 的开发与应用书 [M]. 福州: 福建科学技术出版社, 2013: 49.

[11] 高树中, 杨骏. 针灸治疗学 [M]. 北京: 中国中医药出版社, 2012, 8: 148.

[12] 郭帅良, 廖穆熙, 贺君, 等. 基于中医传承辅助平台的针灸治疗中风后肩手综合征选穴组方规律分析 [J]. 中国中医急症, 2019, 28(11): 1899 – 1902.

[13] 杨波, 宋丽鼎, 王小雪, 等. 电针治疗膝关节骨性关节炎的临床研究 [J]. 双足与保健, 2019, 28(18): 151 – 152.

[14] 牛凤菊, 石冬梅, 刘旭军. 阳陵泉穴的临床应用 [J]. 针灸临床杂志, 2003, 19(11): 41 – 42.

[15] 宋京英. 血海穴临证应用举隅 [J]. 中国针灸, 1994(S1): 251 – 252.

[16] 张少珍, 陈治忠. 梁丘穴应用举隅 [J]. 针灸学报, 1992(1): 25 – 26.

[17] 刘伟, 刘云龙, 吕天元, 等. 运用恢刺法治疗“筋伤”验案举隅 [J]. 按摩与康复医学, 2020, 11(7): 56 – 57.

[18] 庞军, 唐宏亮, 杨扬, 等. 推拿足少阳胆经抗衰老思路探析 [J]. 广西中医药, 2007, 30(5): 37 – 39.

[19] 徐湘亭. 论《素问》开阖枢与《太素》关圖枢在意义上的差别 [J]. 江苏中医杂志, 1983(2): 4 – 5.

[20] 邢航, 彭万年. 大杼穴“主膝痛不可屈伸”之理论新议 [J]. 中国针灸, 2017, 37(9): 997 – 999.

[21] 何桢, 吴追乐. 从督脉理论探析大杼的强骨通督作用 [J]. 贵阳中医学院学报, 2017, 39(1): 1 – 4.

[22] 谢晓隽, 裴景春. 悬钟穴临床应用浅析 [J]. 中国中医基础医学杂志, 2011, 17(8): 902 – 903.

[23] 张广梅, 洒玉萍, 赵协慧, 等. 针刺及穴位注射对骨质疏松症模型大鼠生殖内分泌的影响 [J]. 国医论坛, 2020, 35(3): 56 – 58.

[24] 刘农虞. 深刺犊鼻、内膝眼穴治疗膝骨关节炎临床观察 [J]. 上海针灸杂志, 2013, 32(10): 857 – 858.

[25] 张银娟, 杨志新. “相对穴”阴陵泉与阳陵泉治疗关节疾病特异性研究概况 [J]. 河南中医, 2016, 36(7): 1263 – 1265.

[26] 王相奇, 蒋亚芳, 秦克枫. 斜刺血海、梁丘治疗膝关节疼痛 282 例 [J]. 中国针灸, 2003(5): 62.

[27] 李宝岩, 谷忠悦. 合谷穴治疗痛证的整理研究 [J]. 中医临床研究, 2012, 4(12): 121 – 122.

(收稿日期: 2021 – 04 – 06)

(C)1994-2022 China Academic Journal Electronic Publishing House. All rights reserved. http://www.cnki.net
